# Effectiveness of Hepatitis B Vaccination Campaign in Italy: Towards the Control of HBV Infection for the First Time in a European Country

**DOI:** 10.3390/v14020245

**Published:** 2022-01-26

**Authors:** Tommaso Stroffolini, Filomena Morisco, Luigina Ferrigno, Giuseppina Pontillo, Giuseppina Iantosca, Valentina Cossiga, Simonetta Crateri, Maria Elena Tosti, the SEIEVA Collaborating Group

**Affiliations:** 1 Department of Tropical and Infectious Diseases, Policlinico Umberto I, 00161 Rome, Italy; tommaso.stroffolini@hotmail.it; 2 Gastroenterology and Hepatology Unit, Department of Clinical Medicine and Surgery, University of Naples Federico II, 80138 Naples, Italy; filomena.morisco@unina.it (F.M.); giu.pontillo@gmail.com (G.P.); valentina.cossiga@gmail.com (V.C.); 3 National Center for Global Health, National Institute of Health (Istituto Superiore di Sanità ISS), 00161 Rome, Italy; giuseppina.iantosca@iss.it (G.I.); simonetta.crateri@iss.it (S.C.); mariaelena.tosti@iss.it (M.E.T.)

**Keywords:** HBV, Italy, epidemiology, risk factors, intravenous drug users

## Abstract

Background: In 1991, a mass immunization campaign against the hepatitis B virus (HBV) for children and teenagers was introduced in Italy. This study evaluated the impact of the immunization campaign on the incidence and modes of HBV transmission. Method: Acute HBV cases of viral hepatitis were reported to the National Surveillance System (SEIEVA). Hepatitis A cases reported to the same system were used as controls to calculate the adjusted odds ratios and the population attributable risk for potential risk factors. Results: The incidence of acute HBV declined from 5.0 in 1990 to 0.4 in 2019 per 100,000 population. The fall was almost total in people targeted by the campaign: in 2019, zero cases (100% reduction) in the age-group 0–14 years and 0.1 cases per 100,000 population (99.4% reduction) in the age-group 15–24 years were reported. In the decade 2010–2019, nearly one-fifth (19.3%) of cases occurred in foreigners. Intravenous drug use is no longer a risk factor (OR = 0.7; 95% CI = 0.5–1.02). Beauty treatments, risky sexual exposure, and household contact with an HBsAg carrier were found to be independent predictors of acute hepatitis B. Conclusions: The HB vaccination campaign proved effective in minimising acute HBV in Italy. Control of the infection is close to being reached for the first time in Europe.

## 1. Introduction

In 1991, Italy was the first European country to introduce mandatory vaccination against the hepatitis B virus (HBV) for 3-month-old infants and for 12-year-old children (limited to the first 12 years of the campaign for the latter category). In 1992, the program became fully operative. In 2004, the vaccination of 12-year-old adolescents ended, whereas the program for infants was maintained. As consequence, all subjects <40 years of age are currently vaccinated against HBV. Immunization of teenagers was an important target because it afforded protection before the onset of risk-taking behaviors such as sexual activity and drug use, which are more likely to start in the teenage years.

The vaccination program also included the mandatory screening of pregnant women for the hepatitis B surface antigen (HBsAg), in order to administer active-passive immunization to newborns of positive mothers. In addition, it recommended the vaccine for groups at high risk of infection (including households of chronic HBsAg carriers and intravenous drug users).

This immunization campaign has reached millions of children, with an immunization rate of 95% [1]. In this context, HBV vaccine not only prevents the spread of the virus, but generally reduces the global burden of HB-associated disease. For these reasons, a pivotal role in the combat of HBV infection is held by the HBV vaccine, as further suggested by a recent report [2].

Data from the national surveillance system of acute viral hepatitis (SEIEVA) have previously shown the effectiveness of the vaccination policy in reducing the incidence of acute hepatitis B in Italy in the period 1991–2005, mostly in the age groups targeted by the campaign (a 50-fold decrease in the subjects aged 0–14 years and a 20-fold decrease in the those aged 15–24 years) [3,4].

We report herein the impact of the HB vaccination campaign on the incidence rate of acute HBV after three decades, and on changes, if any, in transmission pattern.

## 2. Materials and Methods

### 2.1. Study Population and Data Collection

SEIEVA is an enhanced surveillance system coordinated by the Italian National Institute of Health and established in 1985 [5]. The number of Italian health units that voluntarily participate in the system has progressively increased, reaching 82.4% of the Italian population under surveillance in 2019.

Case definition and data collection procedures used within the SEIEVA surveillance system are described elsewhere [5]. During the study period, the surveillance methods and notification system were unchanged.

### 2.2. Incidence Rates Estimate

In order to estimate the incidence rates for each type of hepatitis and for each year, the number of the new reported cases was used as the numerator, and the denominator was the population of the local health units participating in the surveillance system.

### 2.3. Case Control Study

To estimate the association linking acute HBV cases with characteristics of subjects and potential sources of exposure, and the population attributable risk (PAR) to various exposures, cases of hepatitis A reported during the same time period to the SEIEVA were used as controls.

### 2.4. Statistical Analysis

To evaluate the statistical significance of the differences observed between groups, chi-squared test or chi-squared for trend was used for proportion, and Mann–Whitney or Kruskal–Wallis test was used for continuous variables. A *p*-value < 0.05 was considered to be significant. A multiple logistic regression model was used to evaluate the independent effect of the considered risk factors on HBV infection; socio-demographic characteristics were considered as adjustment variables.

The population attributable risk (PAR) was calculated according to Levine’s formula [6]: P × (r − 1)/P × (r − 1) +1, where P is the proportion of the general population (in this case acute HAV cases) with the characteristics or exposure, and r is the odds ratio.

## 3. Results

### 3.1. Incidence Data

**Figure 1:**
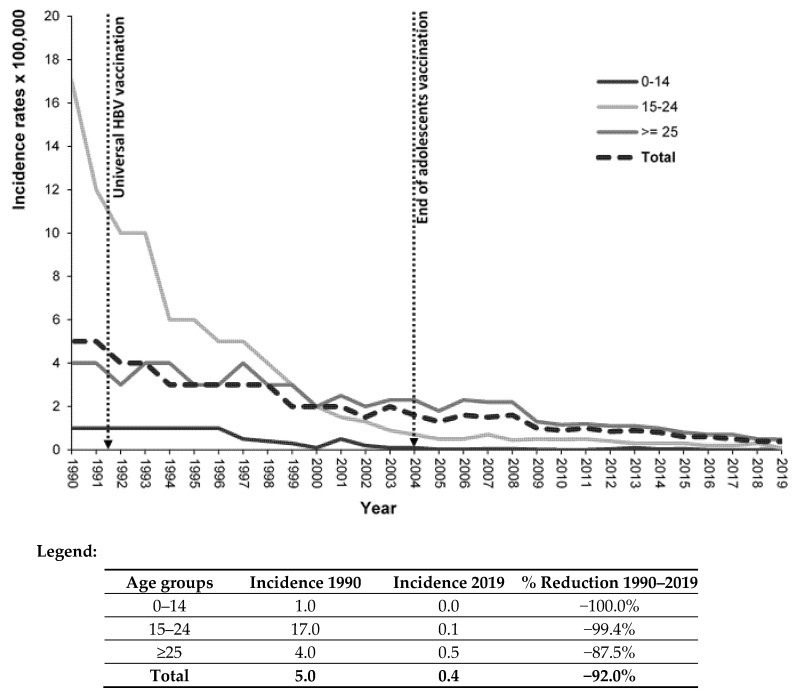
Incidence rates (number of cases × 100,000 inhabitants) of acute hepatitis B in Italy (total and by age group). SEIEVA 1990–2019.

**Figure 2:**
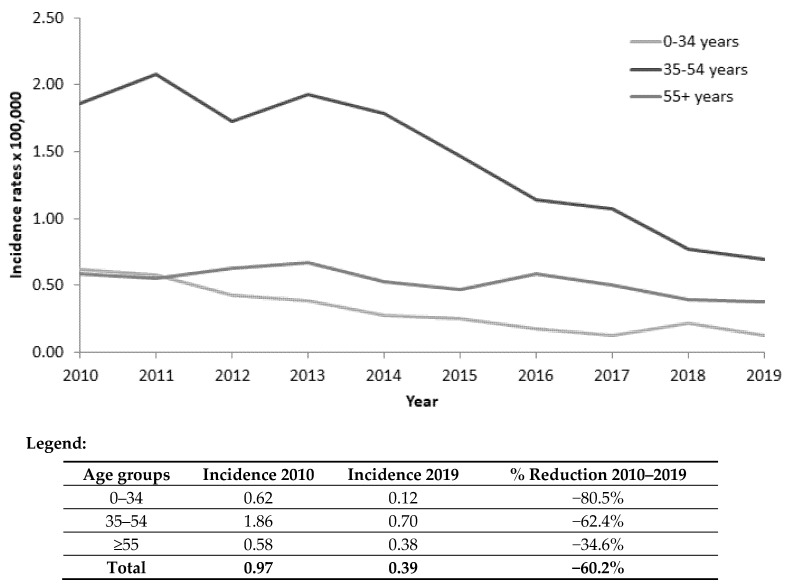
Age-specific incidence rates (number of cases per 100,000 inhabitants) of acute hepatitis B in Italy. SEIEVA 2010–2019.

The overall incidence rate of acute hepatitis B cases per 100,000 inhabitants decreased from 5.0 in 1990 to 0.4 in 2019 (92.0% reduction). The decrease was nearly total in the age group targeted by the vaccination campaign: 0 cases in the age group 0–14 (100% reduction) and 0.1 cases per 100,000 population (99.4% reduction) in subjects 15–24 years old (Figure 1). In the last decade, the decrease was evident even in older age groups (Figure 2).

### 3.2. Main Characteristics

Table 1 reports the features of acute HBV cases by decade. The median age of cases rose from 29 years in the period 1991–1999 to 45 years in 2010–2019 (*p* < 0.01). The proportion of cases having higher years of education, diagnosis in northern/central areas, and escaping mandatory vaccination significantly increased over time (*p* < 0.01). Nearly one-fifth of cases in the decade 2010–2019 occurred in people born abroad. The median number of days spent in hospital decreased from 16 to 10 days (*p* < 0.01); the case-fatality rate increased from 0.5% to 1.1% (*p* < 0.001).

The frequency (%) of non-mutually exclusive risk factors reported in HBV cases by decade (Table 2) highlights that beauty treatments and risky sexual behavior were the most frequent sources of exposure. Intravenous drug use went down from 20.8% in the decade 1991–1999 to 3.3% in 2010–2019 (*p* < 0.01). Blood transfusion without surgical intervention was stable under 1%.

**Figure 3:**
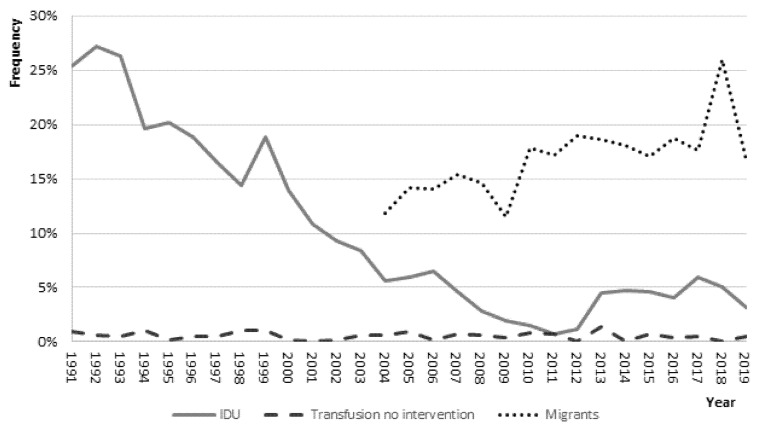
Frequency (%) of some non-mutually exclusive risk factors reported in acute hepatitis B cases by year. SEIEVA 1991–2019.

Figure 3 reports the yearly trends of some characteristics of the subjects: the proportion of people reporting intravenous drug use shows a dramatic decline over time from 25.4% in 1991 to 3.2% in 2019; conversely, acute HBV among foreigners increased over time, peaking at 26% in 2018.

In the last decade (2010–2019), acute hepatitis B in subjects born abroad, as compared to Italian cases, was more likely in subjects who were of female sex (sex ratio 2.0 vs. 3.4; *p* < 0.001), younger (median age 32 years vs. 47 years; *p* < 0.01), and part of households with an HBsAg positive carrier (15.5% vs. 7.4%; *p* < 0.01), but less likely to report risky sexual behavior (22.9% vs. 29.5 *p* = 0.003) (Table 3).

### 3.3. Risk Factor Analysis

Using acute hepatitis A cases reported by the same surveillance system as a control group, the adjusted OR for the association of acute HBV cases with the characteristics of subjects and reported risk factors in the decade 2010–2019 were evaluated by multiple logistic regression analysis (Table 4). All the considered characteristics, except IDU (OR = 0.7; CI 95% = 0.5–1.02) proved to be independent predictors of acute HBV. Blood transfusion was confirmed to be an efficient mode of transmission (OR = 5.08; 95% CI = 1.8–14.3); however, only 16 HBV cases reported such exposure (Table 4).

Population attributable risk (PAR) estimates indicate that 10.2%, 9.1%, and 7.6% of acute HBV cases may be explained by beauty treatments, risky sexual behavior, and household contact with an HBsAg positive case, respectively. Only 1.1% of cases are due to blood transfusion (Table 5).

## 4. Discussion

The present findings highlight the effectiveness of the HB vaccination campaign on the incidence of acute hepatitis B in Italy. The overall decrease in the infection over time was 92%; most important, acute symptomatic HBV infection results were nearly eliminated in the age groups targeted by the campaign: zero cases in subjects 0–14 years old and 0.1 per 100,000 in those 15–24 years old. In the last decade, the favorable impact of the vaccination is evident even in the older age groups, reflecting the reduced spread of the virus.

The decreasing incidence figures are in line with the results of surveys showing the effectiveness of HB vaccination in preventing chronic liver disease (CLD) and HBsAg carriage among the young Italian generation. The mean age of CLD HBsAg-positive cases has shifted from 30.8 years in the decade 1980–1989 [7] to 57.3 years in 2019 [8]. In 2010, no subject below 30 years of age tested anti-HBc-positive in a study performed in a small southern Italian town [9]. In another study during the years 2008–2009 focusing on 13,008 pregnant women, the HBsAg prevalence rate was 0.4%, but no woman younger than 30 years of age (i.e., those targeted by vaccination) tested HBsAg-positive [10].

Taken together, all these findings indicate a current minimal spread of HBV infection in Italy, where control of the virus is close to being reached for the first time in a European country. 

The key explanation for this favorable condition may be the peculiar model of the HBV vaccination policy adopted in Italy. The combined immunization of 3-month-old infants and 12-year-old subjects (limited to the first 12 years of the campaign for the latter category) generated an early immune cohort of youths, who are at high risk of acquiring HBV infection.

Other countries where comprehensive HB vaccination programs were adopted also reported successful results. In Taiwan, children born before and after the introduction of universal HBV vaccination showed a dramatic decrease in the HBsAg carrier rate from 10% in 1984 to 0.9% in 2012 [11]. Moreover, a dramatic declining trend in liver-related mortality was observed after the introduction of the national vaccination program: 68% decline in mortality from fulminant hepatitis in infants and a 75% decrease in the incidence of HCC in children 6–8 years old [12]. Universal newborn vaccination has eliminated HCC and symptomatic HBV infection among Alaskan native children [13]. In Gambia, infant vaccination has achieved substantial protection against chronic HBsAg carriage in early adulthood [14].

A changing pattern is observed in the main modes of HBV transmission. Intravenous drug use, the strongest independent predictor of acute HBV in the period 1995–1997 (adj. OR = 33.1%; 95% CI = 20.4–53.6) [3], is no longer associated with acute infection in the last decade (OR = 0.72; CI 95% = 0.51–1.02). Moreover, a dramatic downtrend in cases reporting this source of exposure from 20.8% in 1991–1999 to 3.3% in 2010–2019 is observed. This finding is different from the corresponding figures in other developed countries; in the USA, despite an overall decline in acute HBV cases attributable to successful vaccination programs, a rise in cases related to drug use has been reported in some states [15]. Further insight into the role of intravenous drug use as an important source of HBV infection in the USA has been recently provided [16]. The prevalence of the hepatitis B core antibody (anti-HBc) in serum (the marker for any previous exposure to HBV) among USA-born individuals has remained flat (around 2.5%) since 2007, coincident with a nearly doubled prevalence from 35.3% in 2001–2006 to 58.4% among people reporting previous intravenous drug use [16].

Taken together, all these findings reflect the reported decline in vaccine uptake among USA-born children and adolescents aged 6–18 years from 1999 to 2016 [17], leaving open the opportunity for new infections in young people.

Our findings might reflect the avoidance of sharing drug use equipment and/or the low proportion of IVDU still susceptible to HBV infection due to already being exposed or previously vaccinated. This latter point seems to be the most likely explanation. Among the 96 acute HBV cases reporting IDU in the decade 2010–2019, as many as 91.1% were unvaccinated subjects. Higher vaccine coverage may further decrease the rate of infection in this risk group, already targeted by the vaccination campaign.

Despite the fact that blood transfusion without surgical intervention continued to be an efficient mode of HBV transmission (OR = 5.1; 95% CI = 1.8–14.3) even in the decade 2010–2019, it accounts for only 1.1% of all HBV cases according to the PAR estimates, as only 16 subjects reported this source of exposure. This favorable finding reflects the introduction in 2008 of nucleic acid testing (NAT) for HBV in blood donor screening, capable of detecting infections in the window phase as well as the presence of occult infections that could potentially be transmitted. A recent survey [18] showed that the overall residual risk of HBV amounts to only 1 in 2,566,854 donations. Moreover, the risk of acquiring HBV infection could also be related to the hospital setting more than to the transfused blood [19].

Cohabitation with a chronic HBAg+ carrier results in the most efficient mode of transmission (OR = 10.8; 95% CI = 7.8–14.9). Alarmingly, rejection of the opportunity for immunization is present in this group, as more than 40% of these subjects, aware of the carrier condition of their cohabitant, were not vaccinated despite the strong recommendation of immunization.

Risky sexual behavior and beauty treatments continue to be efficient modes of HBV transmission. The use of HAV cases as a control group has likely generated an underestimate of the association of sexual behaviors with HBV infection (OR = 2.38; 95% CI = 2.06–2.74), because of an HAV epidemic among homosexual people that occurred in Italy, as well as in several other European countries, during 2016–2017 [20].

According to the PAR estimates, those two risk factors may explain nearly one-fifth of all HBV cases occurring in Italy. Once again, the importance of condom use in cases of casual sexual intercourse and proper sterilization of instruments in beauty treatment settings need to be strongly recommended.

HBV infection in migrant populations represents a new challenge for Italy and the rest of Europe; in fact, migrants may face a higher risk of contracting communicable diseases because they are often exposed to various risk factors before, during, and after migration. Moreover, instability and disruption of national healthcare services in their countries of origin may lead to suboptimal vaccine coverage at arrival [21]. As a consequence, most of them are still susceptible to infection, and, once they have acquired HBV, they may maintain the spread of the infection. Currently, they represent nearly one-fifth of all HBV cases occurring in Italy and are three-fold (95% CI = 2.5–3.6) more likely to acquire HBV than Italian natives. Testing and active proposal of HB vaccine to migrants entering Italy represents a useful and advisable preventive measure [22].

A potential limit of the present study is that incidence rates of acute HBV herein reported underestimate the true incidence of the infection, due to the voluntary nature of reporting cases and occurrence of subclinical infections. However, the incidence trend of the infection should not be affected by this limit, as the system was unchanged during the study period. The use of hepatitis A cases as a control group to estimate the odds ratios and the PAR might raise concern, because of differences between HAV and HBV cases by age, sex, and geographical area. The use of multiple logistic regression analysis likely removed the potential confounding effect of these socio-demographic variables. Moreover, comparability between cases and the control is the crucial point in a case-control study, and this is ensured by the fact that, in this study, hepatitis A and B cases were both identified by the same surveillance system, and thus exposed to the same selective factors, if any. Thus, reliable comparison between cases and controls was obtained in this survey. Despite the fact that using HAV cases cannot be considered as absolutely the best control, it may represent a feasible choice in this setting.

Finally, the high proportion of the Italian population covered by SEIEVA, with a nationwide reach, provides representativeness to the observed findings of acute HBV in Italy.

In conclusion, the current spread of HBV infection in Italy is minimal, reflecting the effectiveness of the vaccination campaign. Vaccination coverage of the households of chronic HBsAg carriers and of migrants may accelerate the road towards control of the virus for the first time in a European country.

These features, even if generated in Italy, may also be of interest for other developed countries.

## Figures and Tables

**Table 1:** Main features of acute hepatitis B in Italy by decade. SEIEVA 1991–2019.

Characteristics	1991–1999 N = 6443 *n* (%)	2000–2009 N = 5526 *n* (%)	2010–2019 N = 3148 *n* (%)	*p*-Value for Trend
Sex ratio (M/F)	2.8	3.1	3.0	0.044
Age distribution:				
≤25	2354 (36.7)	596 (10.9)	174 (5.5)	<0.001
26–40	2483 (38.7)	2743 (50.3)	909 (28.9)	
41–60	1063 (16.6)	1616 (29.6)	1617 (51.5)	
≥61	516 (8.0)	502 (9.2)	443 (14.1)	
Median age (range)	29 (1–98)	37 (0–100)	45 (0–98)	<0.001
Years of education:				
≤8	3489 (71.2)	1977 (53.9)	737 (48.4)	<0.001
≥9	1414 (28.8)	1691 (46.1)	784 (51.6)	
Area of diagnosis:				
North/central	5295 (82.2)	4915 (88.9)	2918 (92.7)	<0.001
South/islands	1148 (17.8)	611 (11.1)	230 (7.3)	
Area of birth *:				
Italy			2541 (80.7)	-
Abroad			607 (19.3)	
Escaped mandatory vaccination	55 (0.9)	279 (5.2)	280 (9.2)	<0.001
Icterus	5170 (83.5)	4588 (85.4)	2530 (82.7)	0.869
Hospitalization rate	5893 (93.1)	5084 (93.7)	2853 (91.7)	0.050
Median days spent in hospital (range)	16 (1–98)	13 (1–118)	10 (1–84)	<0.001
Death	30 (0.5)	25 (0.4)	33 (1.1)	0.002

* Information available since 2004.

**Table 2:** Frequency (%) of non-mutually exclusive risk factors reported by acute hepatitis B cases according to decade. SEIEVA 1991–2019. (Information was missing in some cases).

Risk Factors	1991–1999 N = 6443 *n* (%)	2000–2009 N = 5526 *n* (%)	2010–2019 N = 3148 *n* (%)	*p*-Value for Trend
Blood transfusion without surgical intervention	43 (0.7)	22 (0.4)	16 (0.6)	
Surgical intervention without blood transfusion	623 (10.4)	622 (12.0)	291 (10.0)	0.013
Surgical intervention and blood transfusion	107 (1.8)	86 (1.7)	26 (0.9)	
Intravenous drug use	1267 (20.8)	378 (7.2)	96 (3.3)	<0.001
Unvaccinated among IDU *	1137 (95.7)	301 (89.9)	72 (91.1)	<0.001
Beauty treatments **	1827 (29.9)	1662 (31.5)	949 (31.8)	0.044
Sexual exposure ***	1601 (30.4)	1642 (32.5)	818 (28.3)	0.174
Household contact of an HBsAg+ carrier	598 (12.9)	421 (10.4)	201 (8.7)	<0.001
Aware of the positivity of the household	181 (47.8)	160 (43.7)	77 (41.8)	0.153

* Information missing in 79 cases in 1991–1999, 43 cases in 2000–2009, and 17 cases in 2010–2019. ** Piercing, tattooing, acupuncture, manicurist/chiropodist attendance, and barbershop shaving. *** Two or more sexual partners or lack of condom use in cases of casual sexual intercourse.

**Table 3:** Comparison of acute hepatitis B cases in Italy by area of birth. SEIEVA 2010–2019.

Characteristics	Italians N = 2541 *n* (%)	Foreigners N = 607 *n* (%)	*p*-Value
Sex ratio (M/F):	3.4	2.0	<0.001
Age:			
≤40	631 (24.9)	452 (74.7)	<0.001
41–60	1474 (58.1)	143 (23.6)	
≥61	433 (17.0)	10 (1.6)	
Median age (range)	47 (0–98)	32 (2–80)	<0.001
Area of diagnosis:			
North/Central	2343 (92.2)	575 (94.7)	0.032
South/Islands	198 (7.8)	32 (5.3)	
I.V. Drug use	64 (2.7)	32 (6.0)	<0.001
Household contact of an HBsAg+ carrier	144 (7.4)	57 (15.5)	<0.001
Sexual exposure *	698 (29.5)	120 (22.9)	0.003

* Two or more sexual partners or lack of condom use in cases of casual sexual intercourse.

**Table 4:** Frequency (%) of characteristics reported by acute hepatitis B and acute hepatitis A (control group) cases and adjusted * odds ratio (OR) for the association of acute hepatitis B with characteristics of subjects. SEIEVA 2010–2019.

Characteristics	HBV N = 3148 *n* (%)	HAV N = 7517 *n* (%)	OR (95% CI)
Sex:			
Females	780 (24.8)	2240 (29.9)	1
Males	2360 (75.2)	5258 (70.1)	1.42 (1.24–1.63)
Age:			
≤40	1083 (34.5)	4917 (65.5)	1
≥41	2060 (65.5)	2591 (34.5)	5.33 (4.70–6.05)
Area of diagnosis:			
South/islands	230 (7.3)	903 (12.0)	1
North/central	2918 (92.7)	6614 (88.0)	1.59 (1.28–1.92)
Area of birth:			
Italy	2541 (80.7)	6416 (85.4)	1
Abroad	607 (19.3)	1101 (14.6)	3.02 (2.54–3.60)
Blood transfusion without surgical intervention	16 (0.6)	7 (0.1)	5.08(1.81–14.3)
Surgical intervention without blood transfusion	291 (10.0)	300 (4.5)	2.25 (1.83–2.76)
Surgical intervention and blood transfusion	26 (0.9)	10 (0.1)	4.76 (2.01–11.3)
Intravenous drug use	96 (3.3)	230 (3.5)	0.72 (0.51–1.02)
Beauty treatments **	949 (31.8)	1581 (23.4)	1.62 (1.43–1.83)
Sexual exposure ***	818 (28.3)	983 (15.4)	2.38 (2.06–2.74)
Household contact of an HBsAg+ carrier	201 (8.7)	71 (1.2)	10.8 (7.88–14.9)

* Adjusted by logistic regression analysis for the confounding effect of all listed variables. ** Piercing, tattooing, acupuncture, manicurist/chiropodist attendance, and barbershop shaving. *** Two or more sexual partners or lack of condom use in cases of casual sexual intercourse.

**Table 5:** Population attributable risk (PAR *) for risk factors in acute hepatitis B cases in Italy. SEIEVA 2010–2019.

Characteristics	PAR
Blood transfusion without surgical intervention	1.1%
Surgical intervention without blood transfusion	5.2%
Surgical intervention and blood transfusion	1.8%
Intravenous drug use	-
Beauty treatments **	10.2%
Sexual exposure ***	9.1%
Household contact of an HBsAg+ carrier	7.6%

* Calculated by Levine’s formula. ** Piercing, tattooing, acupuncture, manicurist/chiropodist attendance, and barbershop shaving. *** Two or more sexual partners or lack of condom use in cases of casual sexual intercourse.

## Data Availability

The data used to support the findings in this study are available from the corresponding authors upon request.
